# Brucine suppresses proliferation and promotes apoptosis of human cholangiacarcinoma cells via the inhibition of COX2 expression

**DOI:** 10.7150/jca.87514

**Published:** 2023-09-04

**Authors:** Qiang Kang, Kai Zheng, Gai-Ming Jiang, Yu-Kai Li, Yu-Bo Liang, Qin Geng, Chun-Hang Qian, Qing-Bo Wang, Zhong-Yin He, Song-Quan Huang, Chen Yang, Jing Li, Yue-Hua Li, Yang Ke

**Affiliations:** Department of Hepatobiliary Surgery, The Second Affiliated Hospital of Kunming Medical University, Kunming, Yunnan 650101, China.

**Keywords:** Intrahepatic cholangiocarcinoma, brucine, proliferation, apoptosis, COX-2

## Abstract

**Aims** The aim of this study was to investigate the anti-tumor efficacy of brucine on intrahepatic cholangiocarcinoma (ICC).

**Methods** ICC QBC939 cells were treated with brucine, cell viability, cell cycle and apoptosis were analyzed using CCK-8 and flow cytometry. The expression of COX-2 and apoptosis related proteins Casp3, Bax and Bcl-2 were detected by Western blot analysis. QBC939 cells were subcutaneously transplanted into nude mice and the mice were injected with brucine intraperitoneally. The expression of Ki67, COX-2 and apoptosis related proteins were detected by immunohistochemical staining and Western blot analysis.

**Results** Brucine significantly inhibited the proliferation and cell cycle progression while promoted the apoptosis of QBC939 cells. The expression of the apoptotic proteins Casp3 and Bax was upregulated, while the expression of Bcl-2 and COX-2 was downregulated in QBC939 cells with brucine treatment. Moreover, the overexpression of COX-2 could antagonize the effects of brucine on QBC939 cells. *In vivo*, brucine inhibited subcutaneous tumor formation in nude mice, and the expression of Ki67, COX-2 and Bcl-2 decreased while the expression of Casp3 and Bax increased in tumor tissues from nude mice with brucine treatment.

**Conclusions** Brucine can significantly inhibit the progression of cholangiocarcinoma *in vitro* and *in vivo*, and the mechanism may be related to the inhibition of COX-2 expression.

## Introduction

Intrahepatic cholangiocarcinoma (ICC) accounts for about 10-15% of all liver malignancies, and is the second common liver malignancies arising from intrahepatic bile ducts epithelial cells 1. Based on the anatomic localization, cholangiocarcinoma can be divided into perihilar cholangiocarcinoma, distal cholangiocarcinoma and ICC. Major risk factors, such as primary sclerosing cholangitis, hepatobiliary flukes, biliary cirrhosis, and Caroli's disease, are associated with ICC development. Despite remarkable progress in the diagnosis of ICC, patients with ICC still have a dismal prognosis 2. The 3- and 5-year overall survival rates and recurrence rates for ICC patients are 30% and 18%, 35% and 70%, respectively. As the lethal cancer type, treatment strategies remain unsatisfactory. Patients are often diagnosed at late stages due to asymptomatic nature of ICC and not amenable to surgical resection which is the most usually used approach for a cure 3. Therefore, understanding molecular mechanisms of ICC has been of momentous interest to promote early diagnosis and treatment of ICC.

Brucine is identified in the seeds of Strychnos nux-vomica and has been used as an anti-inflammatory drug for arthritis and pesticide in terms of its neurotoxicity 4. In recent years, multiple studies have shown that brucine exhibits antitumor effect in numerous cancers 5-7. However, the antitumor efficacy of brucine on ICC remains unknown. This study aimed to evaluate the efficacy of brucine on ICC using both *in vitro* and *in vivo* models, and investigate the underlying mechanism.

## Materials and Methods

### Cell culture

The human cholangiocarcinoma cell line QBC939 was obtained from Qingqi Biotechnology Development Co., Ltd (Shanghai, China). Cells were maintained in Dulbecco's modified Eagle's medium (DMEM) containing 10% fetal bovine serum (FBS) and 1% penicillin and streptomycin and cultured at 37°C with 5% CO_2_ in an incubator. Brucine (Y0001196) was purchased from Sigma-Aldrich (St. Louis, MO, USA).

### Cell transfection

Human cDNAs for Cox-2 were cloned into pcDNA 3.1 to generate pcDNA/Cox-2, followed by sequencing. QBC939 cells were transfected with pcDNA 3.1 control or pcDNA 3.1/Cox-2 using Lipofectamine 3000 (ThermoFisher Scientific, San Jose, USA).

### Cell proliferation assay

Cell proliferation was evaluated by using CCK-8 detection kit (CK04, Dojindo, Japan). About 20,000 cells were seeded into each well of 96-well plates, and then treated with different concentrations (5, 10, 20 μ M) of brucine and 100 μg/mL diclofenac sodium. Then 10 μl CCK-8 reagent was added into each well and incubated at 37°C with 5% CO_2_ for 4 hours. The absorbance at 450 nm was measured by a microplate reader.

### Flow cytometry

For apoptosis analysis, cells were collected and stained by Annexin V-FITC apoptosis detection kit (556547, BD-Biosciences, San Jose, CA, USA), and then detected on a flow cytometer and analyzed using ModFit LT v.3.0 software. For cell cycling analysis, cells were collected and fixed in 75% ethanol at 4 ºC overnight, and treated with RNase A, followed by staining with propidium iodide (PI). The DNA contents in different groups of cells were characterized by a flow cytometer and analyzed using ModFit LT v.3.0 software.

### Western blot detection

Total proteins were extracted from the cells and tumor tissues by using RIPA lysis buffer containing protease inhibitor. Proteins were separated using SDS-PAGE gels and transferred into PVDF membrane. The membrane was blocked using 5% free-fat milk with 0.1% Tween 20 and incubated with primary antibodies including anti-Caspase 3 (#9662, 1:1,000), anti-Bax (#41162, 1:1,000), anti-Bcl2 (#4223, 1:1,000), anti-COX-2 (#4842, 1:1,000), anti-GAPDH (#2118, 1:1,000) (all from Cell Signaling, Cambridge, MA, USA) overnight. Subsequently, the membrane was incubated by horseradish peroxidase (HRP)-conjugated secondary antibodies (#7074, 1:1,000).and visualized using ECL image system.

### Animals

All animal experimental protocols were approved by Institute Ethics Committee. Specific pathogen-free (SPF) nude mice (6-8 weeks old) were purchased from Hunan Slake Jingda Experimental Animal Co., Ltd, and divided into control group and brucine group. QBC939 cells were seeded into right armpit at a density of 2×10^6^ cells per nude mice. As the xenograft tumor volume reached 100 mm3, brucine was injected into the abdominal cavity of nude mice in the brucine group every day for 10 days. Tumor size and body weight were record every day. At the end of the experiment, xenograft tumors were isolated from nude mice for further analysis.

### Immunohistochemical (IHC)

Xenograft tumors of nude mice were fixed with 4% paraformaldehyde, embed in paraffin, and cut into 4-µm slides. After dewaxing and hydration of the slides, endogenous peroxidase was blocked with H_2_O_2_. After retrieving antigen in 10 mmol/L sodium citrate (pH 6.0), non-specific reactions were blocked by 5% bovine serum. Subsequently, the slides were incubated with primary Ki-67 antibody (#34330, 1:1,000) and secondary antibodies (#7074, 1:1,000), stained with diaminobenzidine (DAB) and counterstained with hematoxylin.

### Statistical analysis

Statistical analysis was performed with SPSS 19.0 software. The results shown were the means ± standard deviation (SD). The ANOVA and Student's t test were used for the comparisons. All tests were two tailed, and p<0.05 was considered statistically significant.

## Results

### Brucine inhibited the viability of QBC939 cells

**Fig 1:**
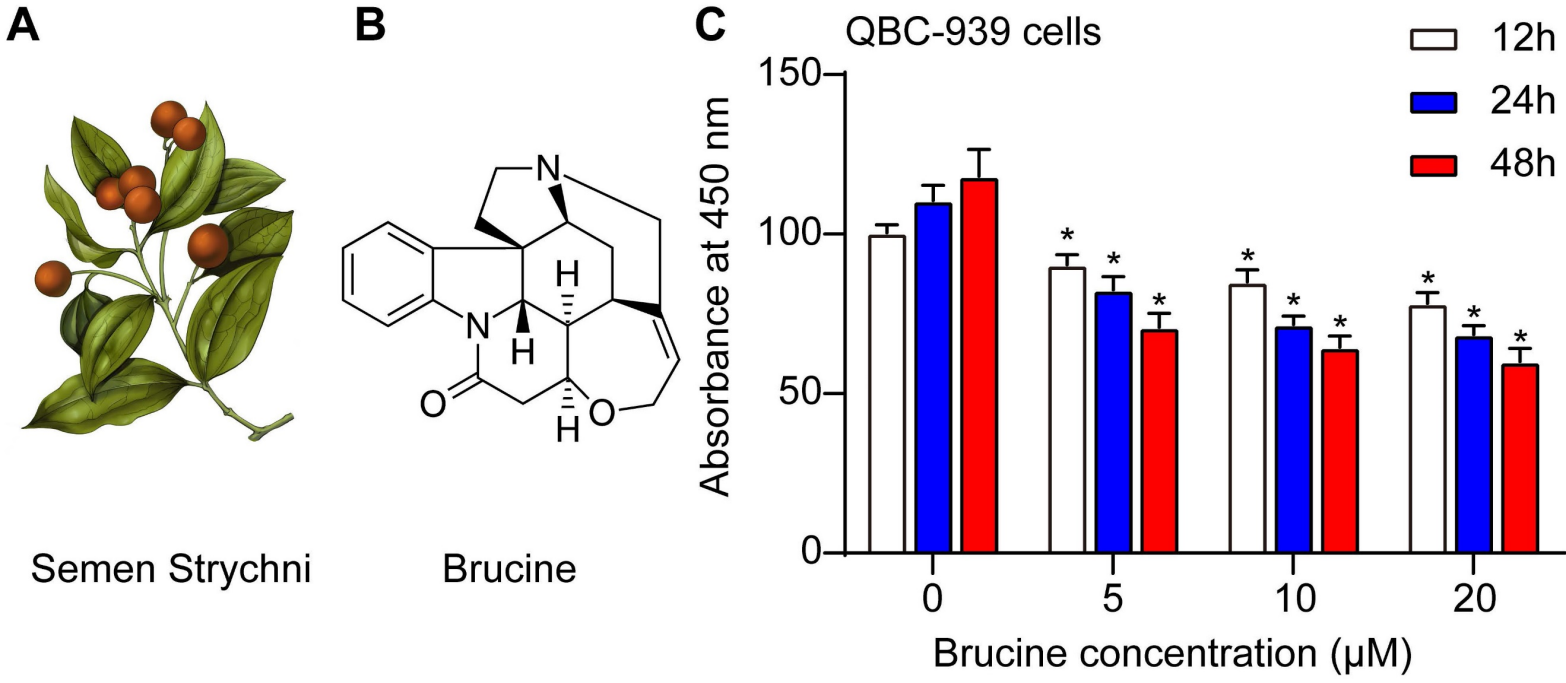
** Brucine inhibited the viability of QBC939 cells.** A. The morphology of Semen Strychni. B. The structure of brucine. C. CCK8 assay of the viability of QBC939 cells treated by brucine. C. Data were expressed as mean ± SD (n=3). *P <0.05.

Fig. 1A showed the morphology of Semen Strychni, and one main component is brucine (structure shown in Fig. 1B). CCK8 assay showed that brucine inhibited the viability of QBC939 cells in dose and time dependent manner (Fig. 1C).

### Brucine promoted apoptosis and inhibited cell cycle progression of QBC939 cells

**Fig 2:**
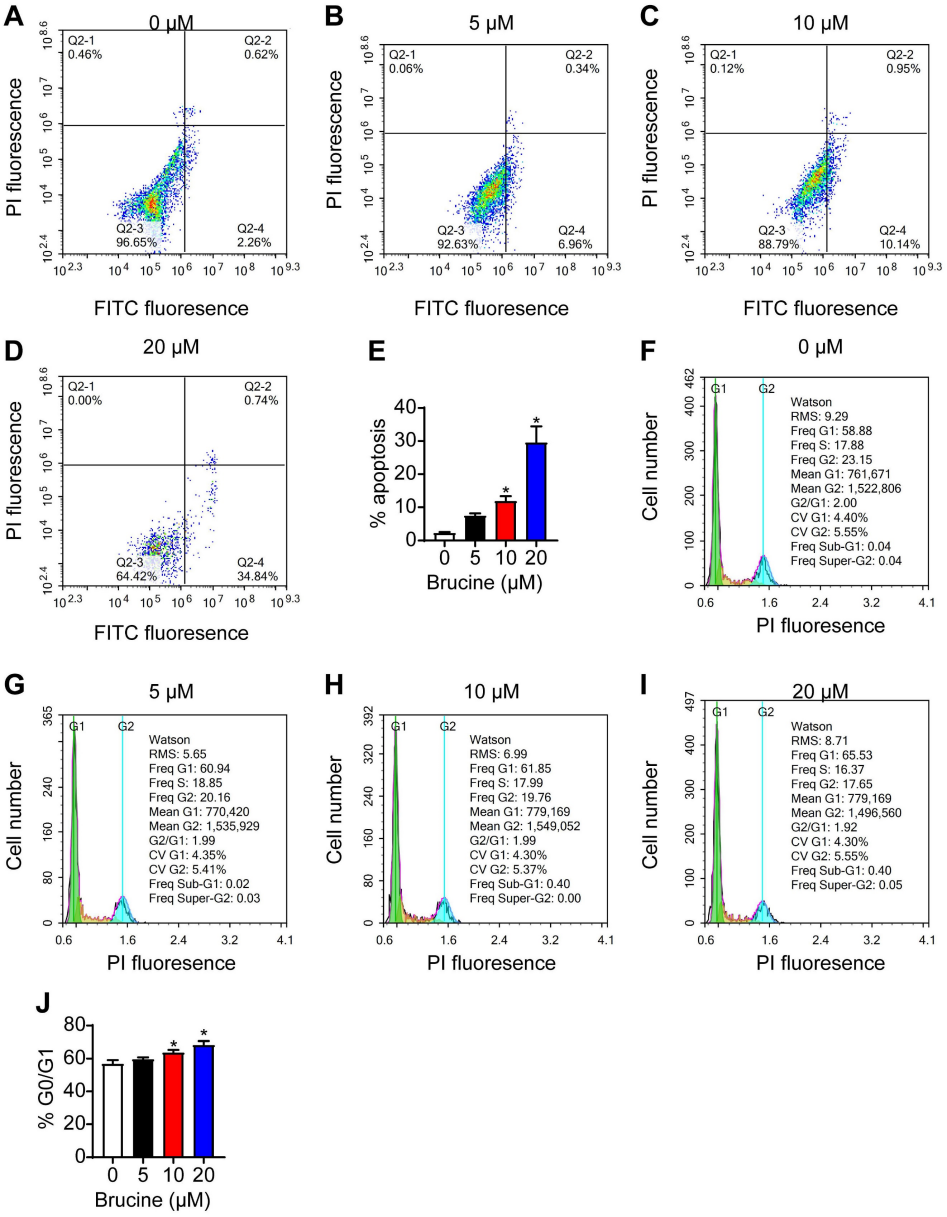
** Brucine promoted apoptosis and inhibited cell cycle progression of QBC939 cells. A-D.** Flow cytometry analysis of apoptotic QBC939 cells. **E.** Quantitative analysis of apoptotic QBC939 cells in each group. **F-I.** Flow cytometry analysis of cell cycle of QBC939 cells.** J.** Quantitative analysis of QBC939 cells in G0/G1 phase in each group. Data were expressed as mean ± SD (n=3). *P <0.05.

Flow cytometry analysis showed that brucine treatment increased the number of apoptotic QBC939 cells in a dose dependent manner (Fig. 2A, B), and increased the number of QBC939 cells at G0/G1 phase (Fig. 2C, D). For the follow-up test, we chose concentration of 10 μM brucine to treat cells for 24 h.

### Brucine promoted apoptosis of QBC939 cells by inhibiting COX-2

**Fig 3:**
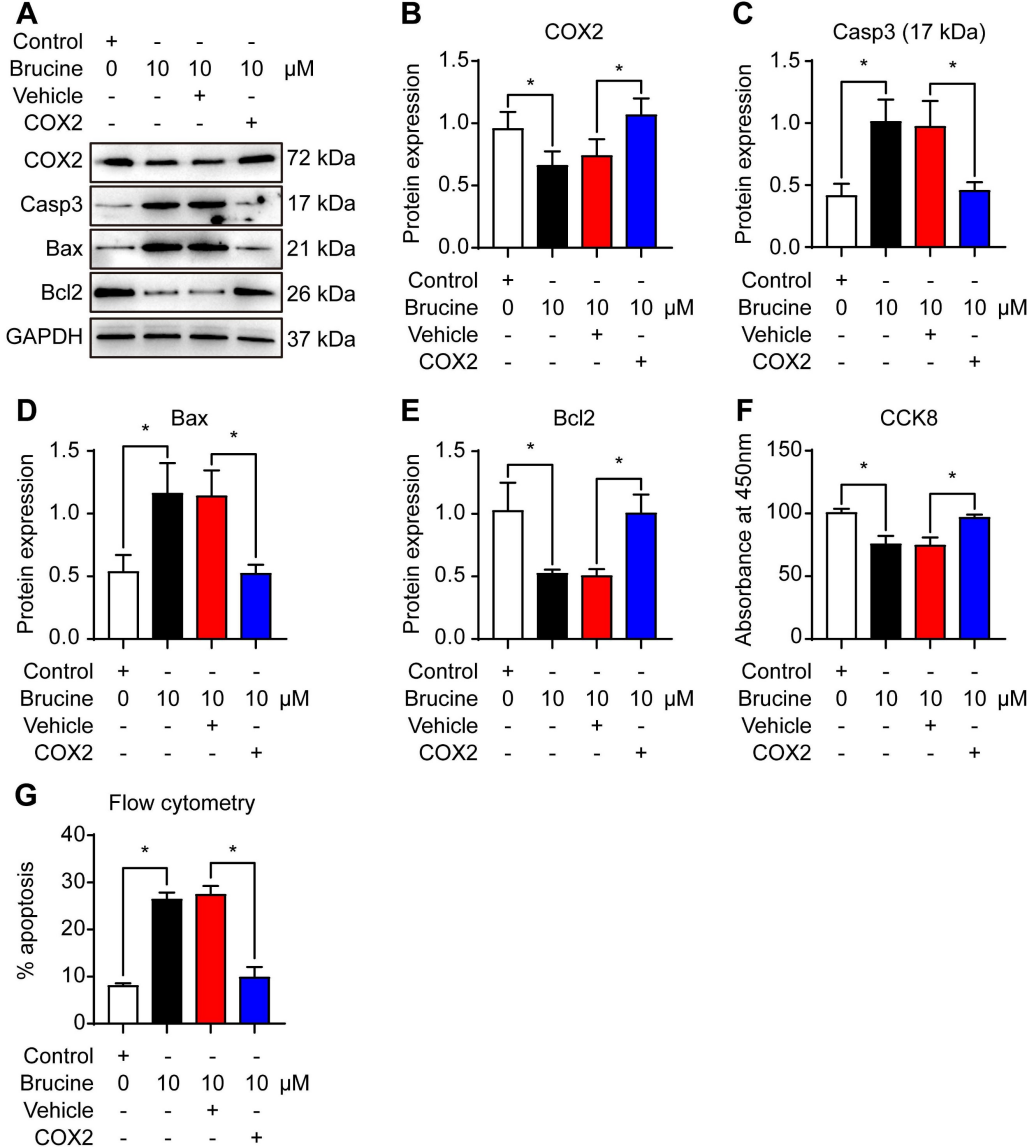
** Brucine promoted apoptosis of QBC939 cells by inhibiting COX-2.** A. Western blot analysis of the expression of COX-2 and apoptosis related proteins in QBC939 cells in each treatment group. GAPDH was loading control. B. Densiometric analysis of COX-2 levels in QBC939 cells in each treatment group. C. Densiometric analysis of caspase-3 levels in QBC939 cells in each treatment group. D. Densiometric analysis of Bax levels in QBC939 cells in each treatment group. E. Densiometric analysis of Bcl2 levels in QBC939 cells in each treatment group. F. CCK8 assay of the viability of QBC939 cells in each treatment group. G. Flow cytometry analysis of apoptotic QBC939 cells in each treatment group. Data were expressed as mean ± SD (n=3). *P <0.05.

To assess the mechanism by which brucine promotes cell apoptosis, we used 10 μM brucine to treat QBC939 cells and/or overexpressed COX-2 and detected the expression of apoptosis related proteins and COX-2 (Fig. 3A). Densiometric analysis showed that brucine inhibited the expression of COX-2 significantly and it was rescued by the overexpression of COX-2 (Fig. 3B). Moreover, brucine increased the expression of caspase 3 and Bax significantly and it was blocked by the overexpression of COX-2 (Fig. 3C, D). Conversely, brucine decreased the expression of Bcl2 significantly and it was rescued by the overexpression of COX-2 (Fig. 3E).

Notably, all these changes of protein expression were consistent with the changes of the viability and apoptosis in QBC939 cells (Fig. 3F, G). These results suggest that brucine promotes the apoptosis and inhibits the proliferation of cholangiocarcinoma cells by inhibiting COX-2.

### Brucine inhibited cholangiocarcinoma cell growth in nude mice

**Fig 4:**
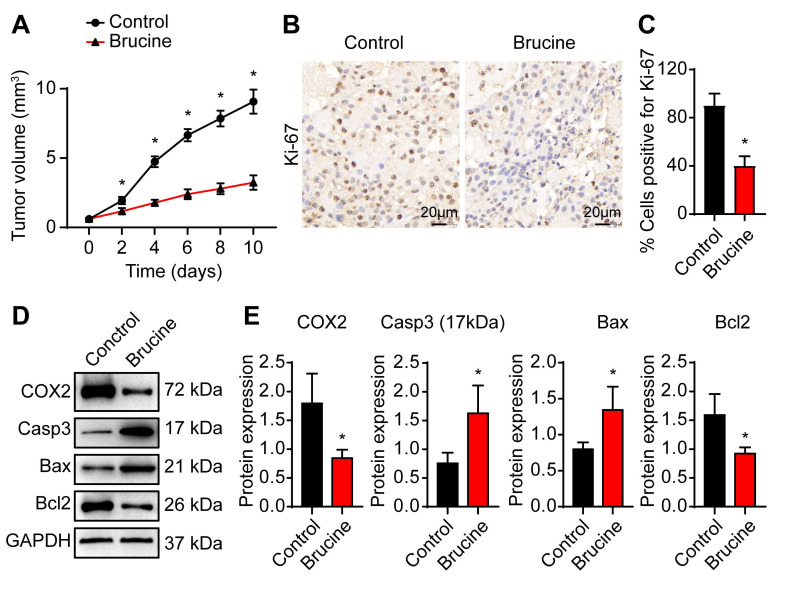
** Brucine inhibited cholangiocarcinoma cell growth in nude mice.** A. QBC939 cells were subcutaneously injected into nude mice After treatment with brucine for the indicated time, tumor volume was measured. B. Immunohistochemical staining of Ki-67 in dissected tumor tissues treated with brucine compared to control group. C. Quantitative analysis of QBC939 cells positive for Ki-67 staining in tissues treated with brucine compared to control group. D. Western blot analysis of the expression of COX-2 and apoptosis related proteins in tissues treated with brucine compared to control group. GAPDH was loading control. E. Densiometric analysis of COX-2, caspase -3, Bax and Bcl2 levels in tissues treated with brucine compared to control group. Data were expressed as mean ± SD (n=3). *P <0.05.

To confirm anti-tumor efficacy of brucine *in vivo*, we subcutaneously injected QBC939 cells into nude mice to establish *in vivo* tumor model. After treatment with brucine, tumor growth decreased significantly in a time dependent manner (Fig. 4A). Immunohistochemical analysis of the dissected tumor tissues showed that Ki-67 staining was significantly weaker after treatment with brucine compared to control group (Fig. 4B, C). In addition, Western blot analysis of the expression levels of COX-2 and apoptosis-related proteins showed that compared with the control group, the expression of Caspase3 and Bax increased and the expression of COX-2 and Bcl2 decreased in mice treated with brucine (Fig. 4D, E).

## Discussion

ICC is one of the primary liver cancers and has highly malignant tumor characteristic. Surgical resection is the most common choice to cure ICC patients in early stages. However, the experience with surgical treatment in ICC is unsatisfactory because of distant metastasis and poor prognosis 8,9. Therefore, the exploration of new therapies is important to improve the prognosis of ICC patients.

Brucine is an alkaloid with bitter taste and high toxicity, and it is derived from the seeds of Strychnos nux-vomica and usually used as anti-inflammatory and analgesic drug. Recent studies have shown that brucine exerted anti-tumor effects in several cancers by different mechanisms. Shu et al. showed that brucine inhibited hepatocellular carcinoma cell migration and metastasis by regulating hypoxia inducible factor 1 pathway 10. For prostate cancer, brucine could inhibit the proliferation of PC-3 cells by inhibiting HSP70 expression and the mitochondrial apoptotic signaling pathway 7. In addition, brucine could suppress colon cancer via the regulation of Wnt/β-catenin and KDR signaling pathways 11,12. Furthermore, recent studies have shown that brucine could regulate ferroptosis and autophagy and could be used for cancer immunotherapy with minimal toxicity 13-15. Therefore, in this study we aimed to explore the benefits of brucine as a novel agent for cholangiocarcinoma therapy. Our results showed that brucine inhibited the viability of QBC939 cells in a dose- and time- dependent manner. In addition, the apoptosis rate of QBC939 cells significantly increased, and cells were blocked at G0/G1 phase after treatment with brucine in a dose- dependent manner. These results indicate that brucine could inhibit the proliferation and promote the apoptosis of ICC cells.

COX-2, an enzyme which could convert arachidonic acid into prostaglandins, plays important role in inflammation and cancer. COX-2 derived prostaglandin at chronic inflammatory diseases may lead to cancer 16. Multiple signaling pathways are activated by COX-2-induced synthesis of prostaglandins in cancer cells, including apoptosis pathway 17-20. High COX-2 expression is detected in a variety of cancers, and is closely related to the characteristics of malignant tumor features and poor prognosis 21. COX-2 could regulate p53 activity and inhibit DNA damage-induced apoptosis 22. Therefore, we postulate that COX-2 may regulate the expression of apoptosis related proteins in cholangiocarcinoma cells via p53 pathway 23. Further studies are needed to explore this possibility.

Therefore, COX2 inhibitors are promising drugs for cancer therapy 24. In this study, we found that brucine can inhibit the expression of COX-2, and regulate the expression of apoptosis related proteins both in QBC939 cell model and in xenograft mouse model. Interestingly, overexpression of COX-2 in QBC939 cells with brucine treatment could reverse the anti-tumor activity of brucine. Therefore, we speculate that brucine exerts an anti-tumor effect on cholangiocarcinoma by inhibiting the expression of COX-2. However, we need use other cholangiocarcinoma cell lines to confirm our conclusion in future studies.

Given the important role of COX-2 in cancer development, COX-2 inhibitors have been developed as novel agents for cancer treatment. However, prolonged use of COX-2 inhibitors could cause life-threatening cardiovascular side effects 25. Recently, nature-derived COX-2 inhibitors emerge as new leads for developing COX-2 inhibitors with less side effects 26. In this aspect, brucine as a natural alkaloid derived from the seeds of Strychnos nux-vomica could be exploited as a novel COX-2 inhibitor for cancer therapy. In conclusion, using both *in vitro* and *in vivo* models we demonstrate that brucine can significantly inhibit the progression of cholangiocarcinoma, and the mechanism may be related to the inhibition of COX-2 expression.
